# Endo metabolomic profiling of *flor* and wine yeasts reveals a positive correlation between intracellular metabolite load and the specific glycolytic flux during wine fermentation

**DOI:** 10.3389/fmicb.2023.1227520

**Published:** 2023-10-19

**Authors:** Charlotte Vion, Mathilde Brambati, Grégory Da Costa, Tristan Richard, Philippe Marullo

**Affiliations:** ^1^Biolaffort, Bordeaux, France; ^2^UMR Oenologie 1366, Université de Bordeaux, INRAE, Bordeaux INP, BSA, ISVV, Paris, France

**Keywords:** phenotypic trade-offs, *flor* and wine yeast, metabolite load, ^1^H-NMR, fermentation kinetics

## Abstract

This study explored the intracellular metabolic variations between 17 strains of *Saccharomyces cerevisiae* belonging to two different genetic populations: *flor* and wine yeasts, in the context of alcoholic fermentation. These two populations are closely related as they share the same ecological niche but display distinct genetic characteristics. A protocol was developed for intracellular metabolites extraction and ^1^H-NMR analysis. This methodology allowed us to identify and quantify 21 intracellular metabolites at two different fermentation steps: the exponential and stationary phases. This work provided evidence of significant differences in the abundance of intracellular metabolites, which are strain- and time-dependent, thus revealing complex interactions. Moreover, the differences in abundance appeared to be correlated with life-history traits such as average cell size and specific glycolytic flux, which revealed unsuspected phenotypic correlations between metabolite load and fermentation activity.

## Key points

1. The phenotypic variability of intracellular metabolites was evaluated by NMR analysis.
2. This revealed differences in the abundance of intracellular metabolites between strains.
3. Metabolic load appeared to be correlated with life history and fermentative traits.

## 1. Introduction

Genetic and enzymatic regulation of metabolic pathways is decisive for the ecological and evolutionary success of living organisms. One well-studied case of such metabolic control is the Crabtree effect, which governs the catabolism of glucose by yeast in a sugar-rich environment (Pronk et al., 1996). The fitness effect provided by the Crabtree effect is well illustrated by the alcoholic fermentation performed by the budding yeast *Saccharomyces cerevisiae* that generally outcompetes other yeast species present in grape juice (Drumonde-Neves et al., 2021). In such conditions, *S. cerevisiae* preferentially produces alcohol from sugar by repressing the use of oxidative respiration, even in the presence of oxygen (Piškur et al., 2006; Merico et al., 2007). This metabolic strategy promotes the fast development of its biomass (Pfeiffer et al., 2001) coupled with ethanol production (Goddard, 2008) and temperature increase (Balsa-canto et al., 2020). Interestingly, ethanol-producing yeasts such as *Saccharomyces* and *Brettanomyces* genera can also efficiently catabolize ethanol at the end of the alcoholic fermentation process (Hagman et al., 2013). This metabolic transition (diauxic shift) has been intensively investigated by molecular approaches (Zampar et al., 2013). The yeast’s ability to switch from ethanol production (fermentation) to ethanol consumption (respiration) is based on the presence of parallel enzymatic reactions, including alcohol dehydrogenase enzymes (Thomson et al., 2005) that occurred after whole genome duplication. This fast production and consumption of ethanol is referred to as the “make-accumulate-consume” strategy (Piškur et al., 2006; Hagman et al., 2013) and might be considered as evidence of the specialization of *S. cerevisiae* for sweet fruits. However, other authors have indicated that this species might be a generalist, making it capable of surviving in a wide range of environments (Goddard and Greig, 2015).

Resource-utilization strategies are intimately linked to metabolic trade-off and are subjected to natural selection. For example, individuals of the same species may take different paths of metabolic adaptation in a changing environment, as recently demonstrated (Ekkers et al., 2022).

The winemaking process constitutes an anthropic niche in which at least two specific populations of the same species may coexist. As demonstrated by several phylogenomic studies, *flor* (velum) yeast and wine yeast are two closely related groups of yeasts with distinct genetic characteristics (Coi et al., 2017; Eldarov et al., 2018; Peltier et al., 2018b). These divergent populations are characterized by genomic signatures in many genes involved in the biofilm formation (*FLO11*), metal transport (*FRE-FIT region* and *ZFR1*), sugar uptake (*HXT3* and *FSY1*), and signaling pathways (*RAG2* and *SFL1*) (Fidalgo et al., 2006; Coi et al., 2017; David-Vaizant and Alexandre, 2018; Eldarov et al., 2018; Legras et al., 2018). Interestingly, *flor* and wine yeast populations allowed the emergence of a specific “Champagne” cluster of wine yeasts resulting from their hybridization. Several quantitative genetics studies demonstrated that the segregation of *flor* and wine alleles in a F1-hybrid progeny clearly impact the phenotypic properties of yeast during alcoholic fermentation. *Flor*-specific alleles were reported to control asparagine uptake (*ASP1*) (Marullo et al., 2007), fermentation kinetics and pH resistance (*PMA1*) (Martí-Raga et al., 2017), glycerol production and malic acid consumption (*PNC1, SDH2, MAE1, PYK2, MSB2*, and *PMA1*) (Peltier et al., 2021). In addition to this genetic evidence, proteomics and transcriptomic analyses depicted the transition of metabolic pathways occurring during the velum formation such as stress resistance, oxidative carbon metabolism, nutrient uptake, protein maintenance, DNA reparation, and cell wall biogenesis (Moreno-García et al., 2015a,b). Complementary information on *flor* yeasts has been compiled in dedicated reviews (Alexandre, 2013; Legras et al., 2016).

However, despite the numerous studies focusing on the specific characteristics of *flor* yeast, comparative physiological studies of *flor* and wine yeast are surprisingly rare (Esteve-Zarzoso et al., 2001; Nidelet et al., 2016; Legras et al., 2018). A metabolic survey of a large panel of *Saccharomyces* strains recently revealed that the final concentrations of malic and succinic acids may be significantly different between *flor* and wine yeast populations (Vion et al., 2023). These organic acids play a central role in the TCA cycles and would be markers of divergent metabolic strategies between *flor* and wine groups. In order to better characterize these possible metabolic features, we aimed to quantify the intracellular content of the most abundant metabolites of the yeast. The quantification of intracellular metabolites is particularly challenging since many environmental disturbances can affect their concentration due to rapid degradation or enzymatic conversion, as reviewed by Pinu et al. (2017). Yeast metabolome has been particularly well investigated and several protocols have been compared for their selectivity regarding different chemical families (Villas-Bôas et al., 2005; Canelas et al., 2009; Kim et al., 2013). According to Villas-Bôas et al. (2005), methanol extraction is the most universal and convenient method since this solvent is able to permeabilize yeast membrane, act as a quenching agent and extract a large variety of intracellular metabolites. Endo-metabolite extraction has been used to investigate the variability of endo-metabolites belonging to different pathways such as amino acids biosynthesis (Hans et al., 2001) and diauxic shift (Zampar et al., 2013). This approach is also useful for understanding the effect of gene expression (Lourenço et al., 2013) and the assimilatory routes of carbon sources (Ogawa et al., 2021). Once extracted, such metabolites can be quantified using two main analytical chemistry approaches, NMR or mass spectrometry (Marshall and Powers, 2017).

NMR presents the advantage of being readily quantitative and requires minimal sample preparation, without the use of preliminary separation techniques. Several studies have demonstrated its relevance for studying yeast metabolome (Lourenço et al., 2013; Ogawa et al., 2021). In recent work, we used basic ^1^H-NMR-based analysis to quantify extracellular metabolites during alcoholic fermentation. In the present study, we implemented this method to compare the endo-metabolome of a restricted number of *flor* and wine strains for the first time.

## 2. Materials and methods

### 2.1. Yeast strains used and culture methods

The strains of *S. cerevisiae* used are listed in Supplementary Table 1. Seven of them belong to the *flor* group while seven others have been clearly characterized as wine yeasts. Three other strains, SB, FMGS_889, and AC1_191, have a mixed genome related to *flor* and wine yeasts. The ISVV-2D strain resulted from a cross between the haploid strains CLIB 1770 and CLIB 1769 delivered by the CIRM collection and derived from the *flor* strain 2D (Coi et al., 2016). *S. cerevisiae* strains were propagated on YPD 2% (1% peptone, 1% yeast extract, and 2% glucose) at 30°C in both liquid and plate cultures (2% agar). Long-term storage at −80°C was achieved by adding one volume of glycerol to YPD overnight cultures.

### 2.2. Alcoholic fermentation assays

#### 2.2.1. Grape juices

The grape juice used for the alcoholic fermentation was a Sauvignon Blanc 2021 (SB21). It was collected in the Bordeaux area and stored at −20°C. Its final composition in terms of fermenting sugar, malic acid content and pH is listed in Supplementary Table 2.

#### 2.2.2. Alcoholic fermentation monitoring

Small-volume alcoholic fermentations were carried out in screwed vials according to the general procedure described in Peltier et al. (2018a). Twenty milliliter-screwed vials (Thermo Fisher Scientific, Bordeaux, France) were filled with 12 ml of grape must and were tightly closed with screw caps (Agilent Technologies, hdsp cap 18 mm PTFE/il 100 pk, Les Ulis, France) and perforated with hypodermic needles (G26-0.45 × 13 mm, Terumo, Shibuya, Tokyo, Japan) to release the CO_2_. The vessel was inoculated by 2.10^6^ viable cell.ml^–1^ precultured in liquid media, 50% filtered must, 50% sterile H_2_O for 24 h. Cell concentration and viability was estimated by flow cytometry (see below). Fermentation took place at 24°C in shaken vials using an orbital shaker (SSL1, Stuart, Vernon Hills, IL, USA) at 175 rpm. Fermentation kinetics was estimated by manually monitoring (1–2 times per day) the weight loss caused by CO_2_ release using a precision balance with automatic weight recording (LabX system, Mettler Toledo, Viroflay, France). The amount of CO_2_ released every hour was modeled from the raw data using non-parametric loess polynomial regression (Peltier et al., 2018a). The computed data allows the estimation of the time necessary to reach the maximum CO_2_ produced as well as the lag phase. The lag phase was determined as the time necessary to reach the first 2 g/L of CO_2_ produced in accordance with the study by Zimmer et al. (2014). The fermentation rate (dCO_2_/dt) was computed by calculating locally the rate of CO_2_ produced hour per hour from the loess dataset. The loess data were also used for calculating the average speed between 15 and 50% of the fermentation (V15_50) considering that on average the trains produced 93 g/L of CO_2_. The exact difference of CO_2_ between the first value with a CO_2_ value higher than 13.95 g/L and the first point of with a CO_2_ value higher than 46.59 g/L. Those data were used to calculate the average speed between 15 and 50% of the fermentation (V15_50). The specific fermentation speed between 15 and 50% (sV15_50) of the fermentation was obtained by dividing V15_50 by the viable biomass collected during the growth phase.

### 2.3. Biomass collection

**FIGURE 1:**
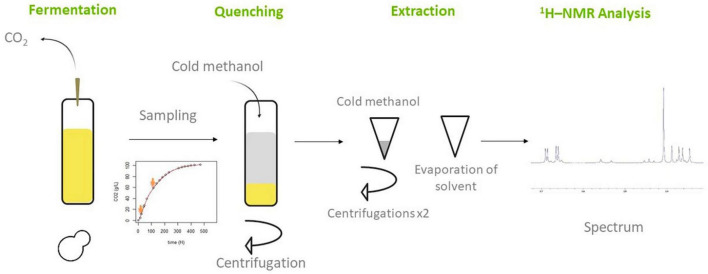
Schematic representation of the experimental steps from fermentation to ^1^H-NMR analysis.

Some vials were set aside at 10 and 50% of the maximal CO_2_ produced for the extraction of intracellular metabolites. Five milliliters were taken per vial for quenching and metabolites extraction. Measurements of cell density, viability and cell size were taken by flow cytometry for each sample (CytoFlex, Beckman Coulter apparatus). Enzymatic assays of malic acid, acetic acid, glycerol, succinic acid, and residual sugars were performed on all samples. Figure 1 summarizes the experimental steps from fermentations to NMR analysis.

#### 2.3.1. Flow cytometry analyses

Flow cytometry measures were taken for each biomass sample and to adjust the cell concentration during inoculation using a CytoFlex (Beckman Coulter, Villepinte, France). Twenty microliter of homogenized culture with 3 μM propidium iodide (PI) were diluted in 1 ml final McIlvaine buffer (sodium phosphate dibasic 0.2 M, citric acid 0.1 M) and was analyzed by flow cytometry (Zimmer et al., 2014). A total of 50,000 events were recorded per experiment. The viability of cells was estimated using 488 nm excitation, with a 610/20 nm long bandpass filter to detect PI fluorescence (dead cells). The Mean Forward-Scatter parameter (Mean FSC) was used to estimate the size of the cells.

The ploidy of the strains was controlled using the method described by Todd et al. (2018). This involved collecting the yeast cells during their growth phase, permeabilizing them with ethanol 70% for 4 days at 4°C and treating them with RNAse A in 50 mM of Sodium citrate buffer, 50 mM, pH = 8.1. The DNA content was quantified using PI diluted in the sodium citrate buffer. The ploidy level was estimated by measuring the intensity of the 630 nm detector and using haploid, diploid, and tetraploid strains as controls, CLIB 1770, GN, and Hirondelle, respectively as previously described by Albertin et al. (2009).

#### 2.3.2. Intracellular yeast metabolites extraction and sample preparation

A sample of 5 ml of fermenting grape juice was taken from the vials and quickly quenched in cold pure methanol with a (1:5) ratio sample/methanol in 35-ml Teflon centrifuge tubes with screw closures (Nalgene, Thermo Fisher Scientific), which were used to prevent metabolite leaks during centrifugation (Villas-Bôas et al., 2005). The tubes were centrifuged at 4,000 *g* for 5 min to remove the medium. The pellet was resuspended in 1 ml of cold pure methanol and placed at −80°C with agitation for 30 min. The tubes were centrifuged at 10,000 *g* for 5 min and the supernatant was stored in a 1.8 ml microtube. The pellet was washed again with 0.5 ml of cold pure methanol, placed at −80°C with agitation for 30 min. The tubes were then centrifuged at 10,000 *g* for 5 min and the supernatant was collected in the same microtube as before. Methanol was evaporated with a SpeedVac vacuum (RapidVap, Labconco, Kansas City, MO, USA) at 32°C and 25% agitation for 4 h. The microtubes were stored at −80°C until NMR analysis.

#### 2.3.3. Metabolites analysis by ^1^H-NMR

##### 2.3.3.1. Sample preparation

Intracellular metabolites were resuspended in 660 μl of phosphate buffer (Na_2_HPO_4_, 0.1 M, pH 7.0) in 90% H_2_O, 10% D_2_O with 1,3-dimethylamylamine (DMMA) 1 mM as an internal standard to achieve a quantitative experiment and TSP to set the spectrum at 0 ppm. The resulting solution was placed into the NMR tube. Since the majority of the intracellular compounds quantified can be found in the extracellular media, the efficiency of the extraction was verified by adding 5 g/L of galactose to the samples prior to methanol quenching. This sugar is not present in grape juices or in *S. cerevisiae* metabolome. Detection of its presence would therefore be indicative of extracellular contamination. As shown in Supplementary Figure 1, less than 0.1% of galactose added (5 g/L) was found in intracellular extracts supplemented with galactose. To ensure that the addition of methanol was able to extract a high percentage of intracellular metabolites, the supernatant containing extracellular metabolites was discarded and the cell pellets were again treated with 1 ml of cold methanol. The percentage of recovery of the first extraction is given in Supplementary Table 3. On average, the percentage of recovery of a single extraction is higher than 85%, except for ethanol and citric acid that are poorly extracted. The entire experimental setup was carried out in accordance with protocols developed by others and proved the efficiency of the cold methanol extraction.

##### 2.3.3.2. NMR spectra acquisition

Spectra were recorded on a 600 MHz Avance III NMR spectrometer (Brucker, Wissembourg, France) operating at 600.25 MHz, equipped with a TXI 5 mm probe with z gradient coils. The measurement of intracellular compounds was performed at 303 K using TopSpin 4.0.8 software (Brucker, Wissembourg, France) and the number of scans was set at 256, resulting in an analysis time of 35 min per sample. The acquisition parameters were set as follows: Free Induction Decay (FID) was collected into a Time Domain (TD) of 64 K data points, with a Spectral Width (SW) of 16 ppm, an acquisition time (AQ) of 3.40 s and a relaxation delay of 5 s per scan. Then 90° pulse calibration was carried out for each sample automatically, and the shimming was set manually in *gs* mode for each spectrum in order to obtain the finest possible line width (lower than 1 Hz). Water suppression was achieved during the Relaxation Delay (RD) using a shape pulse with a multiple-band selective solvent suppression (20 Hz centered on each water signal), with a power level for presentation of 50.37 dB and a shaped pulse for presaturation of 34.83 dB. The FIDs were multiplied by an exponential function corresponding to a 0.3 Hz line-broadening factor prior to Fourier transformation. A manual phase followed by automatic baseline corrections were applied to the resulting spectrum, which was aligned to zero using the TSP signal.

Intracellular compounds were identified based on previous studies (Puig-Castellví et al., 2015), the use of available databases as YMDB (Jewison et al., 2012) and dosed additions made it possible to identify some other compounds. Compounds were quantified by targeted analysis, by the global spectral deconvolution method (GSD) (Cobas et al., 2011), using the simple mixture analysis (SMA) plugin of MestReNova 12.0 software (Mestrelab Research, Santiago de Compostela, Spain).

### 2.4. Enzymatic assay

For each sample taken during the alcoholic fermentation and at the end, a volume of 800 μl was manually transferred in Micronics tubes (Novazine, Lyon, France, ref: MP32033L) and stored at −20°C. Concentrations of the following organic metabolites were measured: acetic acid, glycerol, malic acid, and succinic acid using the respective enzymatic kits: K-ACETGK, K-GCROLGK, K-MAL-116A, and K-SUCC (Megazyme, Bray, Ireland) according to the manufacturer’s instructions. Glucose and fructose were assayed at the end of alcoholic fermentation using the enzymatic method described by Stitt et al. (1989). All the enzymatic assays were performed by a robotic platform at the Bordeaux metabolomics facilities.^1^

### 2.5. Statistical analyses

All the statistical and graphical analyses were carried out using R software (R Development Core Team, 2016) and plots were generated using the base or *ggplot2* packages. All the raw data are given on the Supplementary Table 4.

#### 2.5.1. Multivariate analyses

The heatmap was generated using the *ComplexHeatmap* package. Data were scaled and centered to compare variables that did not have the same order of magnitude.

#### 2.5.2. Analysis of variance

Analyses of variance (ANOVA) were carried out using the *car* package. The phenotypic variability of the 21 intracellular metabolites was estimated by a linear model (LM1) according to the formula (1):


(1) $$y_{i\,j\,k}={\text{time}}_{i}+{\text{group}}_{j}+\text{strain}\,{\left(\text{group}\right)}_{j\,k}+{\text{inter}}_{i\,j\,k}^{2}+e_{i\,j\,k\,l}$$

where *y* is the value of all the variables for a fermentation time *i* (*i* = 1, 2), *j* (*j* = 1, 2) for groups of yeast strains fermented from grape juice. Each group is composed of *k* strains. The factor strain is nested in the factor group and *k* varies between 1:7 and 1:8 according to the number of strains per group. The term inter^2^*_*ijk*_* represents the first order interaction of each factor and ε_*ijkl*_ the residual. When necessary, non-parametric comparison of samples were carried out using the Wilcoxon–Mann–Whitney or Kruskal test with corrected *p*-values (Benjamini–Hochberg method α = 0.05).

## 3. Results and discussion

### 3.1. Experimental design

In this study, a ^1^H-NMR method was developed to quantify the main intracellular metabolites of fermenting yeasts during alcoholic fermentation. We focused our efforts on the characterization of *flor* and wine yeasts that constitute two distinct *S. cerevisiae* populations (Coi et al., 2017; Peter et al., 2018) and share similar ecological niches. As recently demonstrated, wines made by these two groups may have statistical differences for several organic compounds including malic, acetic, and succinic acids (Vion et al., 2023). The genetic material used is listed in Supplementary Table 1. Seven *flor* strains were compared to eight wine strains. In addition, two control strains (FMGS_889 and AC1_191) were included in the experiment. These strains were selected for their opposed malic acid metabolism as previously reported (Vion et al., 2021, 2022). As specified in the methods, yeast cells were collected during alcoholic fermentation and immediately quenched using cold methanol. Samples were taken at two fermentation moments: 10 and 50% of CO_2_. According to previous works, the growth of yeast strains during alcoholic fermentation occurs up to 15–20 g/L of CO_2_ production (Bely et al., 1990; Marullo et al., 2009; Albertin et al., 2011; Nidelet et al., 2016). The samples taken at 10% of CO_2_ were therefore considered to correspond to the growth phase while samples taken at 50% of CO_2_ were considered to correspond to the stationary phase.

Just before sampling, the concentration and the cell size of yeasts were estimated by flow cytometry. To ensure the reliability of the quantification, five biological repetitions were carried out per modality. In addition, a small volume of fermenting juice was frozen for enzymatic metabolic analyses. The experimental design of this work is shown in Figure 1.

### 3.2. Fermentation and yeast growth monitoring

**FIGURE 2:**
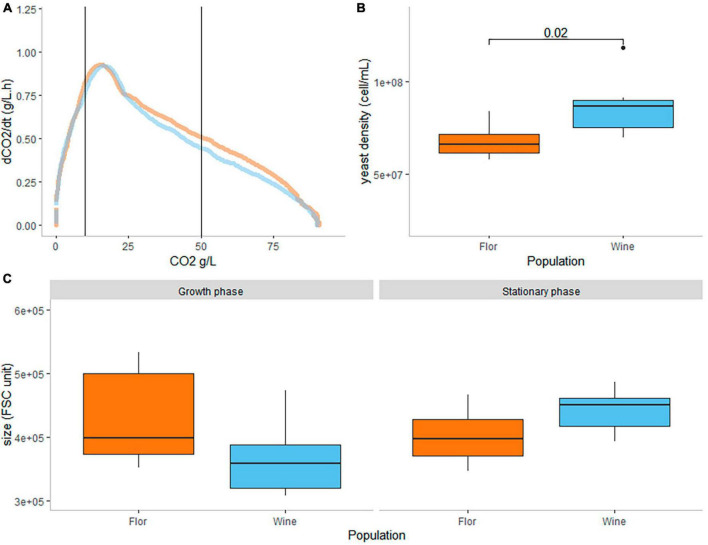
Biomass analysis of yeast cells at the sampling time. **(A)** The time course CO_2_ release of the *flor* and wine strains. Dot lines represent the two sampling points (exponential and stationary phases, 10 and 50 g/L released CO_2_, respectively). **(B)** The cell concentrations at stationary phase (cell/ml) of *flor* and wine yeast were compared (Wilcoxon test, α = 0.05). **(C)** The average yeast size (FSC unit) of *flor* and wine yeast did not evolve in the same way according to the sampling point (ANOVA test, interaction effect α = 0.01).

The average fermentation rate (dCO_2_/dt) of the *flor* and wine groups in function of the CO_2_ produced are shown in Figure 2A. The fermentation kinetics of each individual strain and the CO_2_ production of each culture are provided in Supplementary Figure 2 and Supplementary Table 4, respectively. An important phenotypic variability was observed within groups for the kinetic parameters computed (lag phase, time to reach the maximal CO_2_, and V15_50). Thus, those traits that reflect the fermentation efficiency at the culture level are not statistically different between *flor* and wine yeast in our conditions (Kruskal test, α = 0.05). A similar conclusion has been done in a recent study using other natural grape juice with a subset of the strains used in this study (Vion et al., 2023).

The quantification of cell growth and viability by flow cytometry at two different time courses of alcoholic fermentation allowed the investigation of possible difference in life-history traits between *flor* and wine populations. The average viability of fermenting yeast in the growth and the stationary phases are 92 and 81%, respectively. These traits were not statistically different between *flor* and wine populations. In contrast, the cell concentration reached during the stationary phase is significantly different between *flor* and wine yeasts, with average values of 6.15 × 10^7^ and 8.6 × 10^7^ (Wilcoxon test, α = 0.05) (Figure 2B). The FSC parameter of the flow cytometer was used as a proxy for determining the cell size of both populations. Interestingly, the evolution of cell size between the *flor* and wine groups was statistically different. Wine yeasts were smaller than *flor* yeasts during the growth phase, but they became larger during the stationary phase (Figure 2C), as confirmed by a strong effect of interaction between group and sampling time (two-way ANOVA 21.8% of the variance explained, *p*-value = 0.009).

Since the cell size can be influenced by the ploidy level, we quantified the DNA content of the 15 strains used in this experiment using the method described by Todd et al. (2018). Except for the ISVV-2D strain, all the strains used in this study were found to be diploids, as shown in Supplementary Figure 3. Strain ISVV-2D was obtained by crossing two haploids derivatives of the strain 2D purchased at the CIRM collection; these strains were haploids (with a correct matting type) and were mixed to obtain a diploid clone named ISVV-2D. Intriguingly, the resulting hybrid showed an unexpected ploidy (4n) with G1 and G2 peak intensity close to the tetraploid control (Hirondelle).

### 3.3. Development and optimization of a methodology to extract intracellular metabolites from *S. cerevisiae* and ^1^H-NMR analysis

**FIGURE 3:**
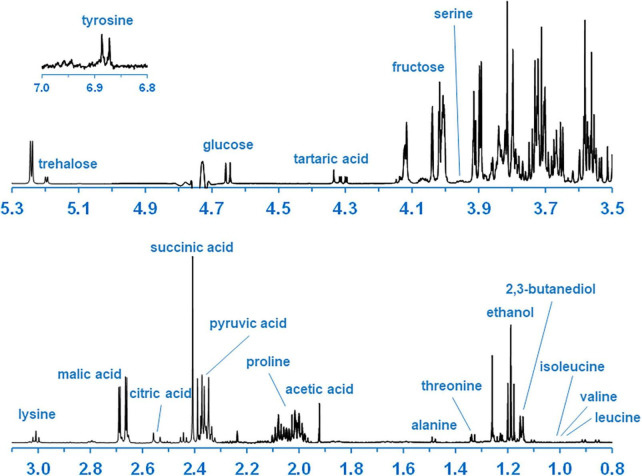
Annotated typical ^1^H-NMR spectrum of intracellular yeasts’ metabolites after water suppression (1D-NOESY-experiment).

The typical ^1^H-NMR spectrum after water suppression is presented in Figure 3. The signals at 0.00 and 1.26 ppm correspond to TSP and DMMA respectively; other signals correspond to intracellular metabolites. The ^1^H-NMR spectra were dominated by sugar content and organic acids followed by some amino acids. The chemical shifts and coupling constant used for identification and absolute quantification of 21 organic compounds are listed in Table 1. The abundance of each compound was normalized by the number of cells per milliliter present in the sample. For both sampling times, the average abundance and CV of each compound and phase are indicated in Table 1. The average abundance of each strain at each sampling point is provided in Supplementary Figure 4.

**TABLE 1:** Chemical shifts and coupling constants used for compounds identification.

Compound	Δ 1 H (multiplicity, J in Hz, assignment)	Growth phase (10% FA)		Stationary phase (50% FA)	
		**CV (%)**	**Abundance (mmol/10^7^ cells)**	**CV (%)**	**Abundance (mmol/10^7^ cells)**
Acetic acid	**1.90** (s, CH_3_)	38.25	1.12 × 10^–04^	33.85	1.76 × 10^–04^
Alanine	**1.50** (d, 7.2, CH_3_)	75.92	8.23 × 10^–05^	38.20	1.34 × 10^–04^
2,3-Butanediol	**1.13** (d, 6.2, 2CH_3_), 3.61 (m, 2CH)	32.73	4.97 × 10^–05^	31.43	2.88 × 10^–04^
Citric acid	**2.54** (d, 15.6, CH_2_), 2.66 (d, 15.6, CH_2_)	27.97	5.45 × 10^–04^	26.40	5.24 × 10^–04^
Ethanol	**1.17** (t, 7.2, CH_3_)	25.32	8.72 × 10^–04^	38.75	1.63 × 10^–03^
Fructose	3.97 (m, CH), **4.01** (dd, 12.8, 1.0 CH_2_), 4.09 (m, 2CH)	22.72	7.73 × 10^–02^	23.59	5.11 × 10^–02^
Glucose	**4.65** (d, 7.9, CH), **5.23** (d, 3.6, CH)	22.36	6.4 × 10^–02^	24.08	2.69 × 10^–02^
Glycerol	3.55 (dd, 11.8 and 6.5, CH_2_), **3.64** (dd, CH_2_)	25.70	3.44 × 10^–03^	24.61	5.39 × 10^–03^
Isoleucine	0.93 (t, 7.4, CH_3_), **1,01** (d, 7.0, CH_3_), 1.24 (m, CH_2_), 1.45 (m, CH_2_), 1.97 (m, CH), 3.66 (d, 3.9, CH)	97.20	1.12 × 10^–08^	54.58	1.83 × 10^–06^
Leucine	**0.96** (t, 2CH_3_), 1.70 (m, CH_3_)	63.17	5.02 × 10^–06^	129.30	6.75 × 10^–06^
Lysine	2,12 (dtd, CH), **3,01** (t, 7.05, CH_2_)	33.74	1.95 × 10^–04^	23.79	2.71 × 10^–04^
Malic acid	2.36 (dd, 16.3 and 7.0, CH), **2.67** (dd, 16.3 and 4.5, CH), 4.30 (dd, CH)	24.19	6.02 × 10^–03^	23.05	5.34 × 10^–03^
Proline	**2.02** (m, CH_2_), 2.06 (m, CH)	29.40	8.38 × 10^–04^	44.33	1.13 × 10^–03^
Pyruvic acid	**2.36** (s, CH_3_)	45.39	8.82 × 10^–05^	29.57	1.44 × 10^–04^
Serine	**3.96** (m, CH_2_)	27.09	2.37 × 10^–03^	23.69	1.64 × 10^–03^
Succinic acid	**2.56** (s, 2CH_2_)	24.42	2.70 × 10^–04^	26.71	9.71 × 10^–04^
Tartaric acid	**4.47** (s, 2CH)	16.35	1.25 × 10^–03^	22.51	1.11 × 10^–03^
Threonine	**1.32** (d, 6.7, CH_3_), 2.58 (d, 4.9, CH), 4.24 (m, CH)	25.15	1.01 × 10^–04^	35.18	2.71 × 10^–04^
Trehalose	**5.2** (d, 3.8, CH_2_)	47.50	3.47 × 10^–03^	35.91	7.71 × 10^–03^
Tyrosine	3.02 (dd, CH_2_), 3.17 (dd, CH_2_), 3.92 (dd, CH), **6.88** (d, 8.4, 2CH), 7.17 (m, 8.6, 2CH)	27.39	1.30 × 10^–06^	32.40	4.10 × 10^–05^
Valine	**0.99** (d, 7.3, CH_3_), 1.04 (d, 7.3, CH_3_), 2.28 (m, CH)	40.77	1.04 × 10^–05^	69.01	1.46 × 10^–05^

The signal chosen for quantification is in bold. Abundance (normalized by the cell number) and coefficients of variation (CV) of intracellular compounds are indicated for both sampling time.

These analytical results demonstrated that the experimental procedure developed can efficiently extract and quantify intracellular metabolites of fermenting *S. cerevisiae*. The extraction methodology was adapted from previous intracellular metabolite extraction protocols. According to Villas-Bôas et al. (2005), cold methanol acts as a non-selective solvent and quenching agent allowing the efficient extraction of different chemical families’ intracellular metabolites. The addition of galactose in the fermentation medium and its absence in the intracellular extracts confirms the efficiency of our protocol (Supplementary Figure 1). Extracted metabolites were then identified and quantified by ^1^H-NMR using the procedures applied in a previous study (Vion et al., 2023).

In order to compare biological samples in the same physiological state, yeast cells were collected according to their progress in the fermentation (10 and 50% of the total CO_2_ expected) during the alcoholic fermentation. These sampling points corresponded to the growth and stationary phases since they occurred before and after the peak of CO_2_ production rate (Figure 2A). The time monitoring of these metabolites may provide information about the evolution of the physiological status of the cells. For example, trehalose is a typical quiescence marker and a key storage carbohydrate (Gray et al., 2004). The abundance of this compound is 2.2-fold higher during the stationary phase in our experiment (Table 1) (Wilcoxon test, *p*-value = 1.14e−08 < 0.05). The detection of this accumulation confirmed the overall physiological changes occurring in fermenting yeasts.

Interestingly, a similar approach was applied to characterize the genetic and environmental factors modulating the intracellular content of *S. cerevisiae* in synthetic media cultures (Airoldi et al., 2015). Compared to this former study, we quantified additional intracellular compounds such as ethanol, 2,3-butanediol, and proline. The detection of additional metabolites would require a higher number of scans and more concentrated biological materials. The analytical parameters are already 256 scans for an analysis time of 35 min per sample. In addition, the evolution of these intracellular compounds was achieved for a larger number of strains (17) compared to Airoldi et al. (only two). By increasing the number of strains, we aim to explore the intraspecific variability of intracellular metabolite abundance that has been poorly investigated so far.

### 3.4. Wine and *flor* yeast showed life history traits and metabolic trade-offs

**FIGURE 4:**
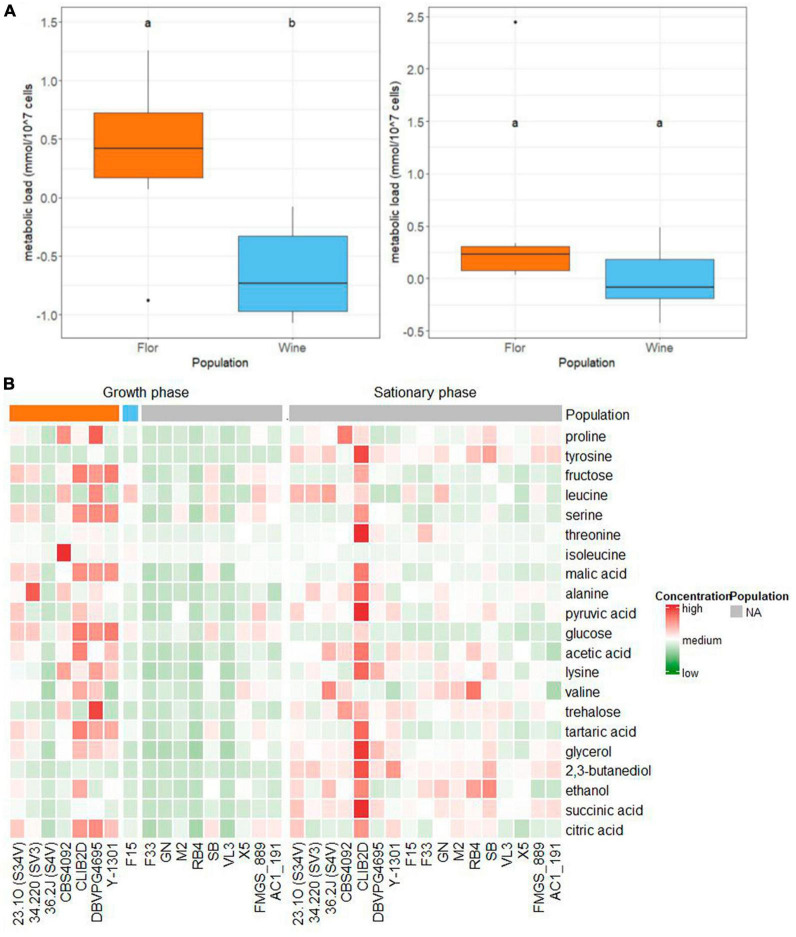
**(A)** Boxplots representing the distribution of the sum of the abundance of each metabolite quantified (cumulated abundance). A Wilcoxon test was applied between the *flor* and wine populations and the resulting *p*-values of the test were annotated on the plot. **(B)** Heatmap of the intracellular metabolites’ concentration per strain. Both sampling times are represented (growth phase and stationary phase). Strains are clustered and colored by population. Concentration values are normalized and expressed as low, medium, or high concentration of metabolites. The control strains AC1_191 and FMGS_889 were also added to the heatmap.

The relative abundance of the 21 intracellular metabolites quantified by ^1^H-NMR was represented by a heatmap according to populations and sampling times (Figure 4). Interestingly, the sum of the abundance of each metabolite quantified is statistically different between *flor* and wine populations during the growth phase. Strains belonging to the wine population displayed a lower pool of intracellular metabolites than strains belonging to the *flor* group (Wilcoxon test, *p*-value = 0.05, Figure 4A). In contrast, no significant differences in the sum of abundance were observed between the two populations during the stationary phase. This surprising finding could reflect a real difference of metabolite concentration inside the cell, or it could be due to biases in abundance normalization. As is routinely done, we normalized NMR intensity by the number of viable cells per milliliter. We alternatively normalized data by the total volume of fermenting yeast that is the product of the cell volume and the number of cells. In both cases, the cumulated metabolite load was significantly higher for the *flor* group, revealing unsuspected variations between *S. cerevisiae* populations during the growth phase of alcoholic fermentation.

Previous studies reported strong correlations between the specific glycolytic flux, the number of fermenting cells and their average size among yeast strains of different origins. These phenotypic trade-offs were reported at different sugar level concentrations (Spor et al., 2008, 2009) and under different alcoholic fermentation conditions (Albertin et al., 2011). The correlation between these life-history traits defined two styles of growth strategies named “ants” and “grasshoppers.” This trade-off reflects the energetic impossibility of making big and numerous cells at the same time in a context of limited nutrient resources. Wine yeasts have been reported to belong to the “ants” group, since they are small, numerous, and ferment quickly at their populational level (Albertin et al., 2011).

**FIGURE 5:**
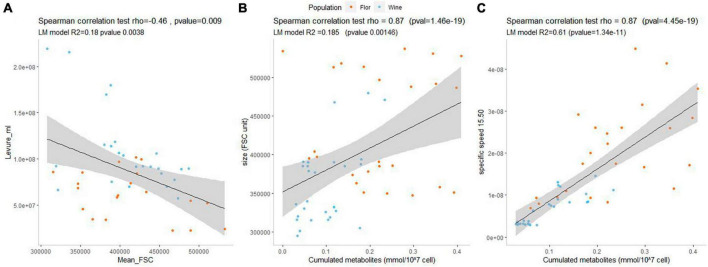
Each dot represents a biomass sample that was quantified by flow cytometry before to be submitted to metabolite extraction. The mean FSC parameter is a proxy of the cell size. **(A)** Correlation between the cell size and the cumulated concentration of intracellular metabolites during the growth phase. **(B)** Correlation between the cell size and the total cell concentration during the growth phase. **(C)** Correlation between the cumulated concentration of intracellular metabolites and the specific glycolytic flux (per cell) during the growth phase. For each panel correlations within variables were tested using the non-parametric correlation test of Spearman.

The quantification of the overall metabolite content of fermenting cells provides the opportunity to test the impact of this new metabolic parameter. First, we confirmed the negative relationship between the cell size and the maximum biotic capacity previously reported by Spor et al. (2008; Figure 5A). In addition, a weak yet highly significant positive correlation (rho = 0.49, *p*-value = 0.0002) was found between the overall metabolite load and the cell size during the growth phase (Figure 5B). Broadly, the biggest cells are those containing the highest intracellular metabolite specific concentration. This trend was only observed during the growth phase when the variation of the cell size between the strains was particularly high (Supplementary Figure 5). Finally, we tested the correlation between the overall metabolite load and the specific glycolytic flux measured between 15 and 50% of CO_2_ released (sV15_50) (Figure 5C). There is a strong and very significative positive correlation between the specific glycolytic flux and the overall metabolite concentration observed in both populations. Strains with a high metabolic load have a higher specific CO_2_ production rate and could be considered as “grasshoppers.”

In addition to the broad differences found between *flor* and wine groups, important strain-specific variations were found. Interestingly, strain SB had the highest relative abundance of intracellular metabolites within the wine population and had the biggest size of the wine yeasts in growth phase. This might be explained by its mosaic origin between *flor* and wine yeasts (Peltier et al., 2021). In contrast, the *flor* yeast 36.2J (S4V) exhibited a low relative abundance of intracellular metabolites, like most strains of the wine group. Other yeast strains displayed distinctive metabolic profiles, such as the *flor* yeast ISVV-2D, which had a much higher content of intracellular metabolites in the stationary phase compared to all other strains. Interestingly, this laboratory-made strain was shown to be tetraploid, which could impact its intracellular metabolite levels. Surprisingly, its average cell size is not particularly higher than that of other diploids *flor* strains, as shown in Supplementary Figure 5. This important strain variability suggested that the cumulated load of metabolites is a complex parameter that remains to be investigated.

### 3.5. The variability of metabolites abundance is mostly characterized by complex genetic × sampling time interactions

**FIGURE 6:**
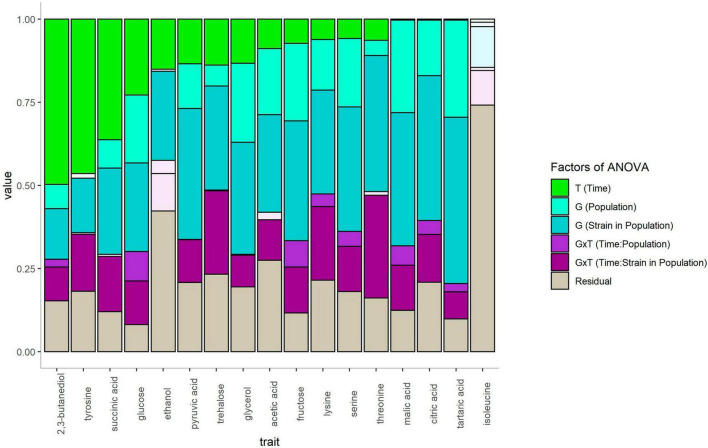
Bar graphs indicating the part of variance explained by the different factors of the ANOVA. The letters T and G represent the time of fermentation and the genetic factors, respectively. The nested ANOVA applied allows to evaluate the effect of the genetic group (population) and the effect of strain within each population (Strain). GxT represents the interaction between genetic and time and was decomposed in two nested factors Time:Population and Time:Strain in Population. Gray tons indicate non-significative effect of the factors.

A nested analysis of variance was applied to deeply investigate the differences in metabolite content between populations and sampling phases. This analysis estimated the effects of the fermentation phase (Time), genetic factors and their possible interactions. The genetic contribution effect was decomposed in population and strain within population effects as detailed in linear model 2 (see section “2. Materials and methods”). The contribution of each factor on 17/21 quantitative traits is summarized in Figure 6. A Levene test (α = 0.05) was performed to test the variance homogeneity and the four traits were excluded from the analysis.

**FIGURE 7:**
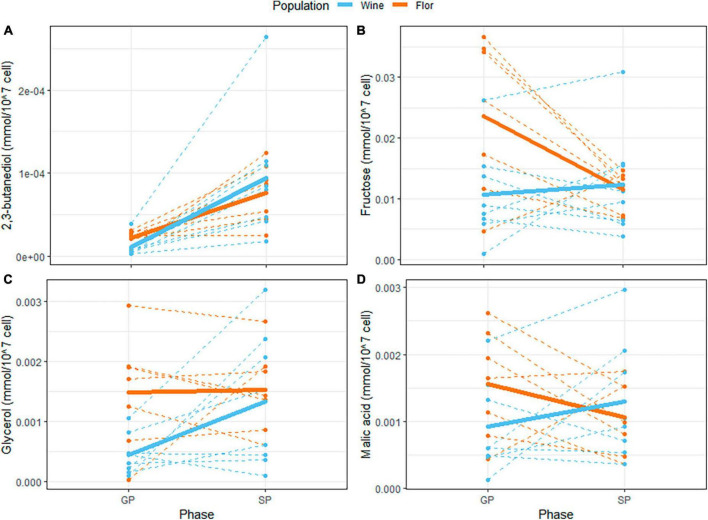
**(A)** Evolution of intracellular 2,3-butanediol content between growth phase (GP) and stationary phase (SP) for the 15 strains. Strains are colored according to their population origin. The bold line represents the average value of the evolution for each population. **(B)** Evolution of intracellular glycerol content between GP and SP. **(C)** Evolution of intracellular fructose between GP and SP. **(D)** Evolution of intracellular malic acid between GP and SP.

Some compounds such as 2,3-butanediol, tyrosine and succinic acid are greatly impacted by the sampling moment, which explained almost 50% of the variance for 2,3-butanediol and tyrosine. Intracellular 2,3-butanediol and tyrosine globally increased four times between growth and stationary phases, while succinic acid was three times more concentrated inside the cells at stationary phase. The accumulation of 2,3-butanediol by yeast cells during stationary phase is shown in Figure 7A. This compound is the end metabolite of the degradation of acetoin, a minor by-product of alcoholic fermentation. Besides these few metabolites, most of the compounds showed quite steady intracellular concentration between the two phases. This phenomenon highlighted that yeast cells are able to maintain relative intracellular homeostasis of their metabolites during the alcoholic fermentation process. This observation also implies that the concentrations measured were not contaminated by the extracellular medium. For example, extracellular fructose is supposed to be drastically consumed between the growth and stationary phases due to the ongoing alcoholic fermentation (40 g/L of CO_2_ produced). However, the intracellular concentration of fructose is quite similar according to the sampling phases (Figure 7B). Interestingly, most of the metabolic compounds (14/17) assayed are strongly impacted by the genetic component, which represented more than 25% of the total variance. Although the population origin was always significative, the decomposition of the population and strain variances indicated a strong strain variability. In addition, an important effect of genetic per time (GxT) interaction was observed for several compounds. This effect ranged between 8 and 31% of the total variance and highlighted the difficulty of establishing a clear pattern of intracellular metabolic content between the *flor* and wine populations, as illustrated for fructose, glycerol, and malic acid (Figures 7B–D, respectively).

Population genomic studies have revealed that the species *S. cerevisiae* is composed of several distinct populations that are characterized by specific genetic and metabolic features. Wine and *flor* yeasts constitute two distinct groups of strains (Coi et al., 2017). Although they share the same ecological niche (wine environment), they developed alternative metabolic strategies for surviving. The first produces ethanol from grape juice while the second consumes ethanol from wine by forming a velum (Legras et al., 2016). Surprisingly few studies have compared the phenotypic properties of wine and *flor* yeast during alcoholic fermentation (Esteve-Zarzoso et al., 2001). In addition, a comparative study of diversity of metabolic networks within numerous *S. cerevisiae* strains encompasses several wine and *flor* strains. The prediction of metabolic fluxes by applying a flux balance analysis suggested that *flor* yeast was characterized by a high production and output of acetate, which contrasted with the behavior of other groups such as bread strains (Nidelet et al., 2016). This discrepancy can be explained by a high variability of accessory metabolic fluxes such as the Pentose Phosphate Pathway. More recently, a metabolomic study identified unsuspected differences in the extracellular composition of wines produced by *flor* and wine strains. Among others, the content of malic acid at the end of alcoholic fermentation clearly discriminated the two groups, with the *flor* group being a stronger consumer of this organic acid (Vion et al., 2023). Since *flor* and wine yeast have evolved under different energy metabolisms (fermentation or respiration) we speculated that they might carry intracellular metabolic hallmarks of these very different selective pressures. The previous section highlighted that the cumulated content of the intracellular metabolites is significantly different between these two groups during the first stages of alcoholic fermentation. Taken individually, none of the metabolites are able to clearly discriminate those groups. Among the metabolites identified by our nested ANOVA as having the strongest impacts, including glucose, fructose, malic acid and citric acid, glycerol has a strong population effect accounting for more than 20% of the total variance explained. However, the strain variability effect within groups is always stronger, making it impossible to define whether those metabolic compounds are group specific.

### 3.6. Comparison of intracellular metabolites abundance of two strains showing opposed malic acid metabolism

**FIGURE 8:**
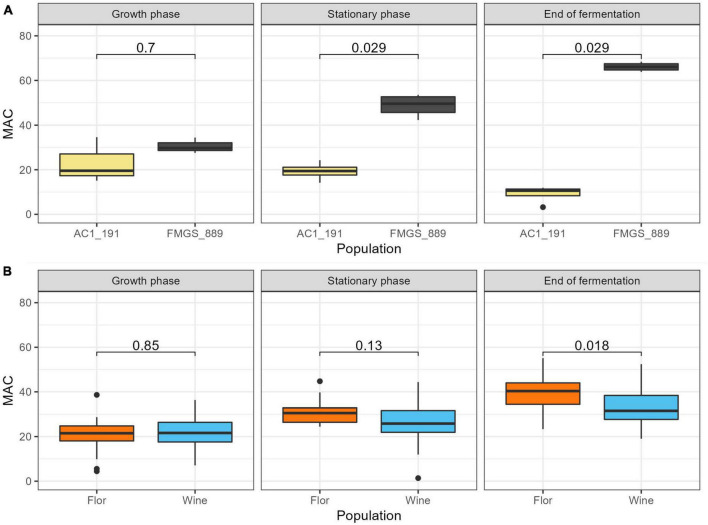
Extracellular malic acid (g/L) according to the fermentation phase. **(A)** A Wilcox test has been performed between AC1_191 and FMGS_889 strains and the resulting *p*-value is displayed on the graphs. **(B)** A Wilcox test has been performed between *flor* and wine populations and the resulting *p*-value is displayed on the graphs.

**FIGURE 9:**
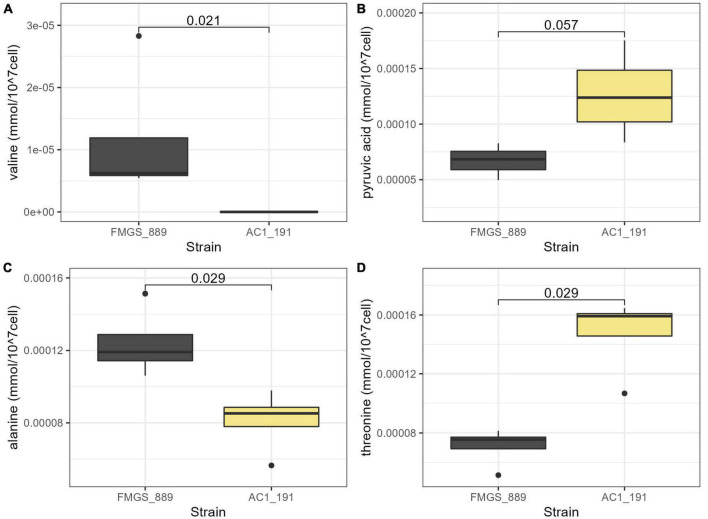
Concentrations of intracellular valine **(A)**, pyruvic acid **(B)**, alanine **(C)**, and threonine **(D)** for the two control strains in stationary phase. A Wilcox test has been performed between the two strains and the *p*-values are displayed on the plots.

The intracellular metabolic profile of the two control strains (AC1_191 and FMGS_889) were compared in this last section. These strains were selected for their strong discrepancy regarding their malic acid metabolism, which is illustrated in Figure 8A. From the stationary phase sampling point, the extracellular malic acid concentration between the two strains is significantly different (difference of 1.23 g/L) and reached 2.4 g/L by the end of alcoholic fermentation. These differences are much higher than those observed between *flor* and wine groups that differ by only 0.15 g/L at the end of alcoholic fermentation (Figure 8B). The NMR quantification method developed provided the opportunity to investigate which intracellular compounds could reflect differences in malic acid metabolism observed at the extracellular level. Surprisingly, the various organic acids quantified showed very similar concentrations between the two strains. Thus, regardless of the malic acid consumption and/or production dynamics, the intracellular concentrations of malic acid and other organic acids (succinic, citric, and pyruvic) are quite steady (Figure 9A). In contrast, the intracellular concentrations of valine, alanine, and threonine measured at the stationary phase displayed a statistical difference between the two strains (Figures 9B–D). These amino acids are indirectly derived from pyruvic and alpha ketoglutaric acid and might result from a difference in malic acid assimilation. However, additional experiments will be needed to establish a metabolic link between these compounds and the malic consumption differences observed between AC1_191 and FMGS_889.

## 4. Conclusion

In this work we developed a ^1^H-NMR protocol to quantify the intracellular concentration of yeast biomass during the alcoholic fermentation of natural grape juice. This protocol was applied to study the phenotypic variability of the metabolic content of several *S. cerevisiae* strains belonging to two distinct genetic populations of the *flor* and the wine yeasts. The precise quantification of 21 metabolites at two time series provided the scientific community with new results. First, the method applied allowed us to detect significant differences between the strains, which demonstrated the variability of the intracellular amounts of the main metabolites. The cumulated concentration of these metabolites is significantly different between the two groups of strains, at least during the growth phase. This difference of abundance is also correlated with life history traits such as average cell size. In addition, we demonstrated that intracellular metabolic variability is governed by the sampling time and the yeast strain with complex interactions that prevent us from drawing simple physiological conclusions. For instance, strains with a very different metabolic production of malic acid are very similar in terms of the majority of their intracellular metabolites. Despite these complex differences, the method and the first results obtained reinforce the interest of this analytic tool for studying the role of intracellular metabolite concentrations in the physiology of the fermenting yeast *S. cerevisiae*.

## Data availability statement

The original contributions presented in this study are included in the article/Supplementary material, further inquiries can be directed to the corresponding author.

## Author contributions

PM and CV: conceptualization, data analysis, and writing first draft. MB, CV, and GD: investigation. CV, PM, and TR: writing and corrections. All authors had read and agreed to the published version of the manuscript.

## Footnotes

1
http://metabolome.cgfb.u-bordeaux.fr/

## Supplementary material

Supplementary Figure 1 NMR spectra showing both glucose and galactose signal in intracellular extracts. The green spectrum has been obtained using the standard extraction procedure with 1 ml cold methanol applied on cell pellet. The red spectrum illustrates the amount of galactose recovered in the extract when 5 g/L of galactose are added just before the quenching step. This compound is not present in the media and constitute a proxy of extracellular metabolite contamination. As shown in the figure only 3.44 mg of this compound was found in the intracellular extract. The blue spectrum illustrates the additional amount of intracellular metabolites that could be recovered by a second methanol extraction. For instance, more than 92% of glucose are extracted by the first methanol extraction.

Supplementary Figure 2 **(A)** Average CO_2_ production over the time. The CO_2_ production was estimated by following the weight loss and converted in g/L of CO_2_ released. Dots represent the average CO_2_ produced of four replicates and was used for applying a loess fit represented by the colored line. The local derivate of the loess fit dCO_2_/dt was calculated and locally smoothed by a mobile mean (*n* = 11) allowing to get the fermentation rate that was plotted again the time **(B)** and the CO_2_ produced **(C)**. The vertical lines indicated the sampling points at 10 and 50% of the alcoholic fermentation.

Supplementary Figure 3 Ploidy level of the 15 strains used in this work estimated by flow cytometry analysis. The strains CLIB1770 and SBE are haploid and tetraploid and were used as control of the ploidy level.

Supplementary Figure 4 Evolution of intracellular content of the 21 metabolites between growth phase (GP) and stationary phase (SP) for the 17 strains. All concentrations are expressed in mmol/10^7^ cell.

Supplementary Figure 5 Each box plot represents the mean FSC parameter considered as a proxy of the cell size for the 15 strains belonging to the *flor* and *wine* groups. The quantification of cell size was shown for the growth and stationary phase on panels **(A,B)**, respectively.
