# Supplementary material for: Endo metabolomic profiling of flor and wine yeasts reveals a positive correlation between intracellular metabolite load and the specific glycolytic flux during wine fermentation
Source: Front Microbiol. 2023 Oct 19;14:1227520. doi: 10.3389/fmicb.2023.1227520 (PMC10620685; doi:10.3389/fmicb.2023.1227520)
Supplement: Supplementary Figure 1 — NMR spectra showing both glucose and galactose signal in intracellular extracts. The green spectrum has been obtained using the standard extraction procedure with 1 ml cold methanol applied on cell pellet. The red spectrum illustrates the amount of galactose recovered in the extract when 5 g/L of galactose are added just before the quenching step. This compound is not present in the media and constitute a proxy of extracellular metabolite contamination. As shown in the figure only 3.44 mg of this compound was found in the intracellular extract. The blue spectrum illustrates the additional amount of intracellular metabolites that could be recovered by a second methanol extraction. For instance, more than 92% of glucose are extracted by the first methanol extraction. [file Data_Sheet_1.docx]

Figure S1


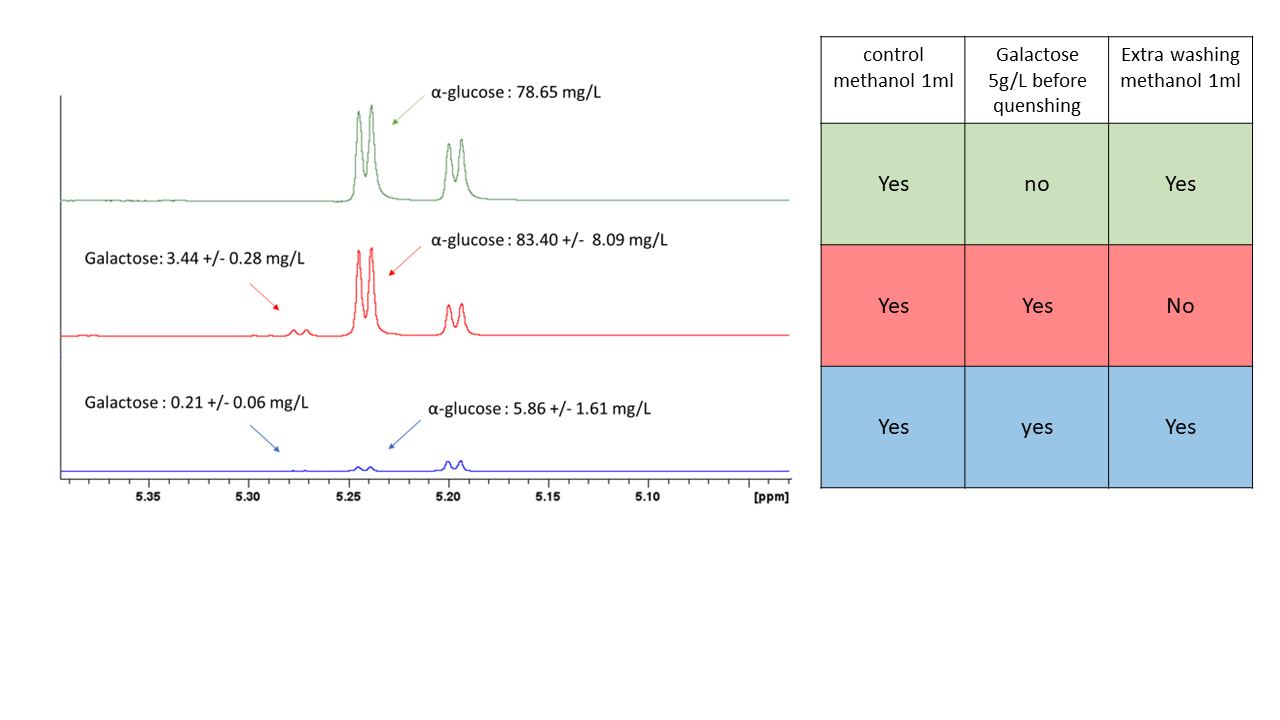


Figure S2


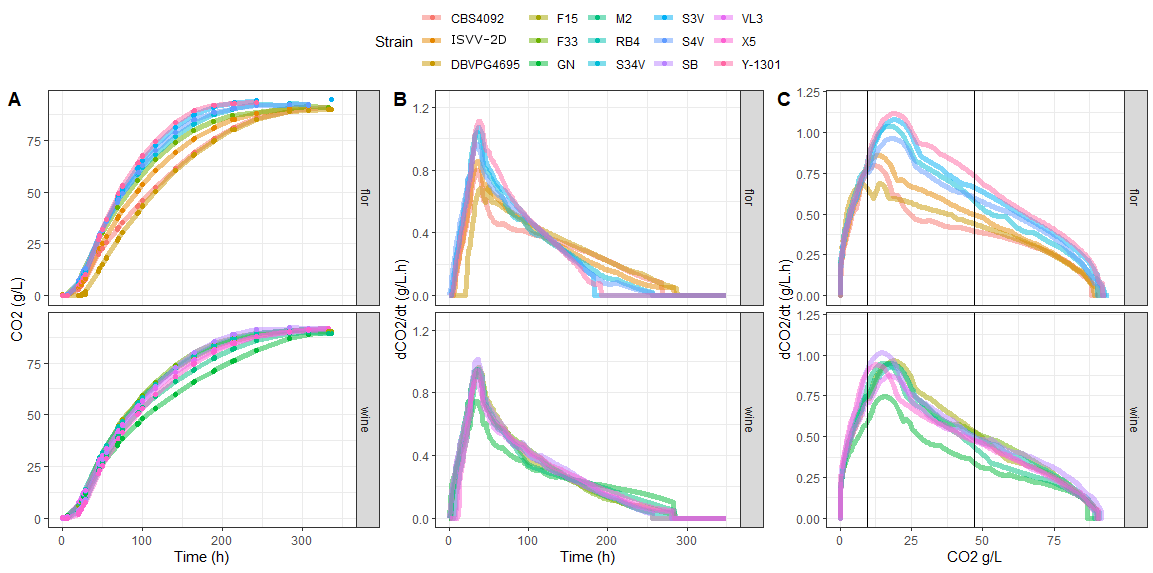


Figure S3

Figure S4


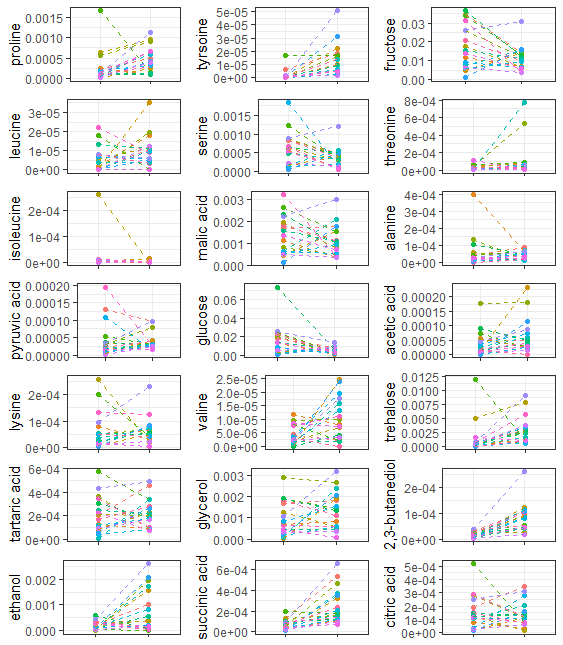


Figure S5


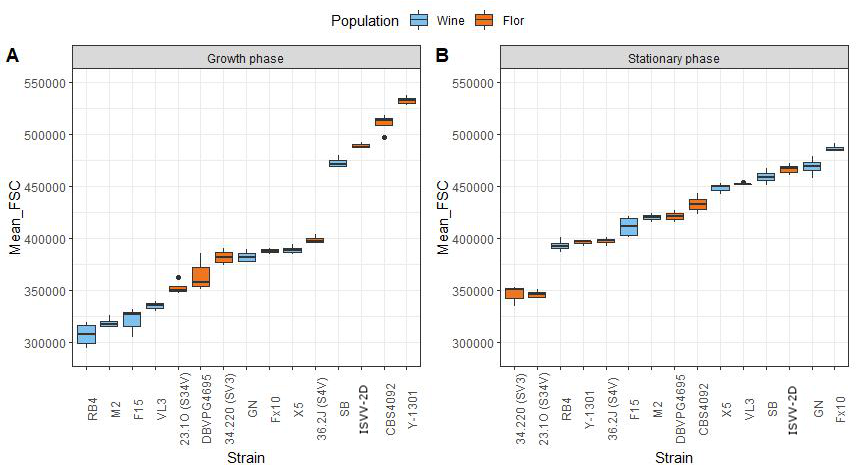


Table S1Yeast strain used

| *Strain* | *Species* | *Ecological niche* | *Substrate/origin* | *Geographical area* | *Collection* | *Reference* |
| --- | --- | --- | --- | --- | --- | --- |
| FMGS_889 | *S. cerevisiae* | Control | Extreme malic acid consuming strain | Miscelaneous (Breeding) | ISVV | (Vion, Peltier, Bernard, Muro, & Marullo, 2021) |
| AC1_191 | *S. cerevisiae* | Control | Extreme malic acid producing strain | Miscelaneous (Breeding) | ISVV | (Vion *et al.,* 2022) |
| 23.10 (S34V) | *S. cerevisiae* | Flor | Velum | Dijon, France | Prof. Hervé Alexandre | (David-Vaizant & Alexandre, 2018) |
| 34.220 (SV3) | *S. cerevisiae* | Flor | Velum | Dijon, France | Prof. Hervé Alexandre | (David-Vaizant & Alexandre, 2018) |
| 36.2J (S4V) | *S. cerevisiae* | Flor | Velum | Dijon, France | Prof. Hervé Alexandre | (David-Vaizant & Alexandre, 2018) |
| ISVV-2D | *S. cerevisiae* | Flor | Velum | Sardinia, Italy | ISVV | This study |
| DBVPG4695 | *S. cerevisiae e* | Flor | Vino Santo | Lungarotti winery, Italy | 1002 genomes | (Peter *et al*., 2018) |
| CBS4092 | *S. cerevisiae* | Flor | Velum | Spain | 1002 genomes | (Peter *et al*., 2018) |
| Y-1301 | *S. cerevisiae* | Flor | Wine | Unknown | NRRL collection | (Vion *et al*., 2023) |
| GN | *S. cerevisiae* | Wine | meiotic spore clone from Zymaflore VL1 | Bordeaux, France | UMR Oenology (ISVV) | (Peltier, Bernard, Trujillo, Barbe, *et al*., 2018) |
| SB | *S. cerevisiae* | Wine | meiotic spore clone from Actiflore BO213 | France | UMR Oenology (ISVV) | (Peltier, Bernard, Trujillo, Barbe*, et al*., 2018) |
| F15msp | *S. cerevisiae* | Wine | meiotic spore clone from Zymaflore F15 | France | UMR Oenology (ISVV) | (Huang, Roncoroni, & Gardner, 2014) |
| M2msp | *S. cerevisiae* | Wine | meiotic spore clone from Enoferm M2 (Lallemand, Canada) | Unknown | UMR Oenology (ISVV) | (Huang *et al*., 2014) |
| VL3 | *S. cerevisiae* | Wine | Wine starter | Bordeaux, France | Laffort |  |
| Fx10 | *S. cerevisiae* | Wine | Wine starter | Bordeaux, France | Laffort |  |
| X5 | *S. cerevisiae* | Wine | Wine starter | Bordeaux, France | Laffort |  |
| RB4 | *S. cerevisiae* | Wine | Wine starter | Beaujolais | Laffort | (Peltier, Bernard, Trujillo, Barbe*, et al*., 2018) |
| CLIB 1770 | *S. cerevisiae* | Flor | Velum | Sardinia, Italy | CLIB | Coi et al. 2016 |
| CLIB 1771 | *S. cerevisiae* | Flor | Velum | Sardinia, Italy | CLIB | Coi et al. 2016 |

Table S2 main enological characteristics of the *sauvignon blanc* grape juice used.

| pH | 3.30 |
| --- | --- |
| L-malic acid | 4.20 g/L |
| Glucose + fructose | 186.1 g/L |
| Assimilable nitrogen | 141 mg N/L |
| Total SO_2_ | 34 mg/L |
|  |  |

Table S3 **% of recovery of the first extraction**

| **Compound** | **% of recovery of the first extraction** |
| --- | --- |
| acetic acid | 87.3 |
| alanine | 80.8 |
| 2,3-butanediol | 78.5 |
| citric acid | 9.1 |
| ethanol | 16.3 |
| fructose | 92.6 |
| glucose | 93.3 |
| glycerol | 87.9 |
| isoleucine | 100.0 |
| leucine | 100.0 |
| lysine | 97.3 |
| malic acid | 87.3 |
| proline | 81.4 |
| pyruvic acid | 96.1 |
| serine | 79.2 |
| succinic acid | 90.0 |
| tartaric acid | 71.1 |
| threonine | 88.6 |
| trehalose | 63.0 |
| tyrosine | 100.0 |
| valine | 56.8 |

Table S4 CO_2_ time course of each culture (Attached csv file)

Table S5

| **Strain** | **Sampling time** | **Malic Acid (g/L)** | **Glycerol (g/L)** | **Acetic Acid (g/L)** | **Glucose (g/L)** | **Fructose (g/L)** | **Succinic acid (g/L)** |
| --- | --- | --- | --- | --- | --- | --- | --- |
| 23.1O (S34V) | 100,0 | 2,5 | 5,1 | 0,4 | -1,0 | 5,0 | 1,6 |
| 34.220 (SV3) | 100,0 | 1,5 | 2,5 | 0,3 | -1,0 | 5,4 | 1,0 |
| 36.2J (S4V) | 100,0 | 2,5 | 5,6 | 0,5 | -1,0 | 4,8 | 1,5 |
| CBS4092 | 100,0 | 1,6 | 3,5 | 0,3 | -0,9 | 5,6 | 0,9 |
| DBVPG4695 | 100,0 | 2,7 | 4,7 | 0,5 | -1,0 | 5,1 | 1,9 |
| F15 | 100,0 | 2,9 | 4,4 | 0,4 | -1,0 | 4,9 | 1,7 |
| FMGS_889 | 100,0 | 1,4 | 4,8 | 0,3 | -1,0 | 5,9 | 1,7 |
| FMGS3_191 | 100,0 | 3,8 | 5,5 | 0,2 | -0,9 | 5,6 | 1,9 |
| Fx10 | 100,0 | 2,6 | 4,7 | 0,4 | -1,0 | 6,1 | 1,5 |
| GN | 100,0 | 2,2 | 3,1 | 0,3 | -1,0 | 5,0 | 1,2 |
| ISVV-2D | 100,0 | 1,5 | 2,8 | 0,3 | -1,1 | 5,3 | 1,2 |
| M2 | 100,0 | 2,7 | 5,2 | 0,3 | -1,0 | 5,6 | 1,7 |
| RB4 | 100,0 | 2,5 | 4,0 | 0,4 | -1,0 | 4,6 | 1,5 |
| SB | 100,0 | 2,5 | 4,4 | 0,3 | 7,5 | 7,9 | 1,3 |
| VL3 | 100,0 | 3,1 | 4,3 | 0,3 | -1,0 | 5,1 | 1,4 |
| X5 | 100,0 | 3,0 | 3,5 | 0,3 | -1,0 | 5,6 | 1,4 |
| Y-1301 | 100,0 | 0,0 | 1,9 | 0,2 | -1,0 | 5,8 | 0,3 |
| 23.1O (S34V) | 10,0 | 3,2 | 3,0 | 0,4 | NA | NA | 0,7 |
| 34.220 (SV3) | 10,0 | 3,2 | 2,4 | 0,4 | NA | NA | 0,5 |
| 36.2J (S4V) | 10,0 | 3,2 | 1,9 | 0,5 | NA | NA | 0,6 |
| CBS4092 | 10,0 | 3,6 | 1,5 | 0,4 | NA | NA | 0,7 |
| DBVPG4695 | 10,0 | 3,5 | 1,8 | 0,3 | NA | NA | 0,6 |
| F15 | 10,0 | 3,3 | 1,5 | 0,4 | NA | NA | 0,6 |
| FMGS_889 | 10,0 | 2,9 | 2,7 | 0,3 | NA | NA | 0,7 |
| FMGS3_191 | 10,0 | 3,3 | 3,0 | 0,3 | NA | NA | 0,7 |
| Fx10 | 10,0 | 3,5 | 1,7 | 0,3 | NA | NA | 0,6 |
| GN | 10,0 | 3,5 | 1,5 | 0,3 | NA | NA | 0,7 |
| ISVV-2D | 10,0 | 3,4 | 1,6 | 0,4 | NA | NA | 0,7 |
| M2 | 10,0 | 3,1 | 2,8 | 0,4 | NA | NA | 0,7 |
| RB4 | 10,0 | 3,2 | 2,3 | 0,3 | NA | NA | 0,6 |
| SB | 10,0 | 3,2 | 2,9 | 0,4 | NA | NA | 0,8 |
| VL3 | 10,0 | 3,4 | 2,0 | 0,4 | NA | NA | 0,6 |
| X5 | 10,0 | 3,1 | 2,1 | 0,4 | NA | NA | 0,6 |
| Y-1301 | 10,0 | 3,4 | 3,5 | 0,6 | NA | NA | 0,5 |
| 23.1O (S34V) | 50,0 | 2,8 | 4,4 | 0,4 | NA | NA | 1,3 |
| 34.220 (SV3) | 50,0 | 2,9 | 3,6 | 0,4 | NA | NA | 1,0 |
| 36.2J (S4V) | 50,0 | 3,0 | 3,6 | 0,5 | NA | NA | 1,1 |
| CBS4092 | 50,0 | 3,1 | 4,1 | 0,3 | NA | NA | 1,0 |
| DBVPG4695 | 50,0 | 3,1 | 3,8 | 0,3 | NA | NA | 1,0 |
| F15 | 50,0 | 2,9 | 3,2 | 0,4 | NA | NA | 1,2 |
| FMGS_889 | 50,0 | 2,2 | 3,9 | 0,3 | NA | NA | 1,2 |
| FMGS3_191 | 50,0 | 3,4 | 3,8 | 0,2 | NA | NA | 1,4 |
| Fx10 | 50,0 | 3,4 | 4,8 | 0,3 | NA | NA | 1,1 |
| GN | 50,0 | 3,4 | 3,6 | 0,2 | NA | NA | 1,1 |
| ISVV-2D | 50,0 | 2,6 | 4,5 | 0,4 | NA | NA | 1,2 |
| M2 | 50,0 | 2,9 | 3,4 | 0,4 | NA | NA | 1,2 |
| RB4 | 50,0 | 2,8 | 4,2 | 0,4 | NA | NA | 0,9 |
| SB | 50,0 | 2,8 | 4,2 | 0,3 | NA | NA | 1,1 |
| VL3 | 50,0 | 3,2 | 3,0 | 0,3 | NA | NA | 0,9 |
| X5 | 50,0 | 3,3 | 3,8 | 0,3 | NA | NA | 1,1 |
| Y-1301 | 50,0 | 2,8 | 4,6 | 0,8 | NA | NA | 0,8 |
